# Volatile Organic Compounds in Uremia

**DOI:** 10.1371/journal.pone.0046258

**Published:** 2012-09-25

**Authors:** Nikolaos Pagonas, Wolfgang Vautz, Luzia Seifert, Rafael Slodzinski, Joachim Jankowski, Walter Zidek, Timm H. Westhoff

**Affiliations:** 1 Deparment of Nephrology, Charité – Campus Benjamin Franklin, Berlin, Germany; 2 Leibniz-Institut für Analytische Wissenschaften ISAS – e.V., Dortmund, Germany; National Cancer Institute, United States of America

## Abstract

**Background:**

Although “uremic fetor” has long been felt to be diagnostic of renal failure, the compounds exhaled in uremia remain largely unknown so far. The present work investigates whether breath analysis by ion mobility spectrometry can be used for the identification of volatile organic compounds retained in uremia.

**Methods:**

Breath analysis was performed in 28 adults with an eGFR ≥60 ml/min per 1.73 m^2^, 26 adults with chronic renal failure corresponding to an eGFR of 10–59 ml/min per 1.73 m^2^, and 28 adults with end-stage renal disease (ESRD) before and after a hemodialysis session. Breath analysis was performed by ion mobility spectrometryafter gas-chromatographic preseparation. Identification of the compounds of interest was performed by thermal desorption gas chromatography/mass spectrometry.

**Results:**

Breath analyses revealed significant differences in the spectra of patients with and without renal failure. Thirteen compounds were chosen for further evaluation. Some compounds including hydroxyacetone, 3-hydroxy-2-butanone and ammonia accumulated with decreasing renal function and were eliminated by dialysis. The concentrations of these compounds allowed a significant differentiation between healthy, chronic renal failure with an eGFR of 10–59 ml/min, and ESRD (p<0.05 each). Other compounds including 4-heptanal, 4-heptanone, and 2-heptanone preferentially or exclusively occurred in patients undergoing hemodialysis.

**Conclusion:**

Impairment of renal function induces a characteristic fingerprint of volatile compounds in the breath. The technique of ion mobility spectrometry can be used for the identification of lipophilic uremic retention molecules.

## Introduction

For centuries physicians have tried to make diagnostic use of specific odors in the air their patients exhale. Hippokrates was one of the first to use the odor of exhaled air for diagnosis [1], [2]. More recently it was Linus Pauling who draw our attention to the fact that the exhaled air is a micro-cosmos of volatile organic compounds (VOCs) [3]. The diagnostic use of exhaled VOCs has been rather modest so far. Although among the various odor qualities of exhaled air “uremic fetor” has long been felt to be diagnostic of renal failure, the VOCs exhaled in uremia attracted little interest. In 1977 Simenhoff et al found that ammonia, trimethylamine and dimethylamine most probably underlie the fishy odor in uremia [4]. However, the exhaled VOCs in renal failure have not been further studied so far, which may be explained by lack of an adequate technique for a systematic global analysis. The present work constitutes the first global analysis of uremic breath using a combination of ion mobility spectrometryand gas chromatography.

There is increasing evidence that lipophilic protein-bound toxins are responsible for several biochemical and functional alterations in uremia [5]. Due to the protein binding the removal of these substances by dialysis is less efficient than for water soluble substances. The retention niveau of lipophilic toxins may still be highly increased although water soluble substances have been successfully removed of the body. The kinetics of urea and creatinine therefore do not adequately reflect the removal of these substances. Hence, there is an ongoing search for alternative diagnostic tools to identify and quantify the retention niveau of lipophilic toxins. The alveolar capillary membrane is most permeable for lipophilic substances, since the membranes of endothelial and alveolar cells are predominantly composed of phospholipids. Therefore primarily lipophilic substances are expected to be exhaled. The present work investigates whether breath analysis may be used for the identification of lipophilic uremic retention molecules.

## Materials and Methods

### Ethics Statement

Written informed consent was obtained from all participants before inclusion in the study. The study was approved by the local ethics committee at the Charité Berlin. All clinical investigation was conducted according to the principles expressed in the Declaration of Helsinki.

### Protocol and study population

Breath analysis was performed by ion mobility spectrometry (IMS) in 28 adults with an estimated glomerular filtration rate (eGFR, calculated according to MDRD formula [6]) ≥60 ml/min per 1.73 m^2^ (group A), 26 adults with chronic renal failure (CKD) corresponding to an eGFR of 10–59 ml/min per 1.73 m^2^ (group B), and 28 adults with ESRD before and after hemodialysis (group C and D). Table 1 provides a characterization of the study population including data on gender, age, body mass index (BMI), cause of renal failure, renal function, comorbidities, and medication. In the ESRD patients, breath analysis was performed in the morning prior to hemodialysis treatment. 22 of the 28 participants with ESRD agreed to repeat the procedure after hemodialysis. Table 2 provides the results of the measurements. Volatile compounds in the breath may be of endogenous or exogenous origin. Therefore, it is crucial that the measurements take place in a standardized, olfactorily “constant” setting. For this reason, all participants were recruited from our internal medicine ward and measurements took place in the same room. The study participants were instructed to refrain from smoking, tooth paste, food/fluid ingestion, and chewing gum for at least two hours prior to the analysis. Moreover, the measurement took place >2 h after the last intake of any kind of medication.

**Table 1:** Study population.

Parameter	Control (n = 28)	CRF, stage 2–4 (n = 26)	CRF, stage 5D (n = 28)
Female	12 (42.9%)	14 (53.8%)	11 (39.3%)
Male	16 (57.1%)	12 (46.2%)	17 (60.7%)
Age	48.8±14.0	68.2±14.6	66.9±11.1
BMI (kg/m^2^)	28.1±7.7	26.7±4.9	24.1±5.6
Creatinine (mg/dl)	0.8±0.2	1.9±0.8	5.1±2.0
Urea (mg/dl)	26.4±8.1	73.0±34.8	106.2±51.0
eGFR (ml/min per 1.73 m^2^)	96.4±22.6	37.2±14.0	<10 (dialysis)
**Comorbidities**			
Diabetes mellitus	4 (14.3%)	6 (23.1%)	12 (42.9%)
Hypertension	13 (46.4%)	22 (84.6%)	26 (92.9%)
Malignoma	1 (3.6%)	0 (0%)	1 (3.6%)
Liver cirrhosis	1 (3.6%)	1 (3.8%)	0 (0%)
**Medication**			
Antihypertensives	12 (42.9%)	22 (84.6%)	25 (89.3%)
Proton pump inhibitors	5 (17.9%)	15 (57.7%)	16 (57.1%)
Statins	2 (7.1%)	9 (34.6%)	7 (25.0%)
Antibiotics	1 (3.6%)	2 (7.7%)	5 (17.9%)
**Cause of renal failure**			
Nephrosclerosis	-	5 (19.2%)	1 (3.6%)
Diabetic nephropathy	-	4 (15.4%)	8 (28.6%)
History of tubular necrosis	-	2 (7.7%)	0 (0%)
Tubulointerstitial nephritis	-	3 (11.5%)	4 (14.3%)
Cystic kidney disease	-	1 (3.8%)	0 (0%)
Glomerulonephritis	-	1 (3.8%)	3 (10.7%)
Urinary tract obstruction	-	2 (7.7%)	0 (0%)
Unknown	-	6 (23.1%)	12 (42.9%)

Classification to stages of chronic renal failure according to the National Kidney Foundation. CRF: chronic renal failure BMI: body mass index. eGFR: estimated glomerular filtration rate, calculated by MDRD formula.

**Table 2:** Analytes with signal intensities indicating renal elimination or association to hemodialysis.

Signal intensity in each group	Peak	Analyte	1/K_0_	t_R_	Δ1/K_0_	Δt_R_	P (ANOVA)
**A<B<C>D**	P1	Hydroxyacetone	0.5341	9.1	0.0041	8.3	0.001
	P2	3-Hydroxy-2-butanone	0.5670	7.1	0.0025	0.6	<0.001
	P3	Ammonia	0.4484	126.4	0.0100	75.7	0.03
	P4	0.5985–55.6	0.5985	55.6	0.0023	4.5	0.03
	P5	0.5468–17.0	0.5468	17.0	0.0041	8.3	0.008
**C, D only**	P6	Heptanal	0.6678	10.5	0.0031	2.8	<0.001
	P7	4-Heptanone Monomer	0.6050	9.5	0.0030	3.2	<0.001
	P8	4-Heptanone Dimer	0.7820	10.4	0.0030	3.2	<0.001
	P9	2-Heptanone	0.6210	11.2	0.0030	3.2	<0.001
	P10	0.6623–10.7	0.6623	10.7	0.0020	2.1	<0.001
	P11	0.5659–11.2	0.5659	11.2	0.0036	1.9	<0.001
	P12	0.6308–11.4	0.6308	11.4	0.0034	3.8	<0.001
	P13	0.7517–10.6	0.7517	10.6	0.0046	2.4	<0.001

Seven of the analytes could be identified by gas chromatography/mass spectrometry (TD-GC/MS). The remaining analytes are described by data of retention time (t_R_) and ion mobility (1/K_0_). Group A: healthy controls; group B: chronic renal failure corresponding to an eGFR of 10–59 ml/min per 1.73 m^2^; group C: end-stage renal disease (ESRD) prior to hemodialysis; group D: ESRD after hemodialysis. Signal intensities were checked for intergroup differences by univariate ANOVA, p<0.05 was regarded significant.

### Ion Mobility Spectrometry (IMS)

**Figure 1:**
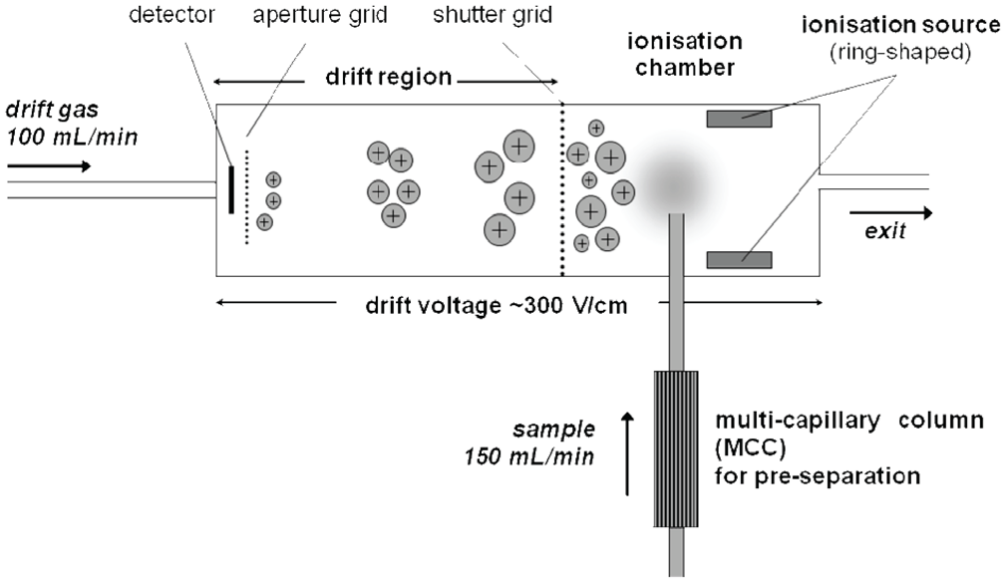
Scheme of an ion mobility spectrometer (MCC/IMS). The multi-capillary column (MCC) provides a preseparation of the molecules in the gas phase. In the ionization chamber proton transfer from the reactant ions to the analyte molecules takes place, thus forming protonated analyte ions. The drift time of the ions in the electric field depends on size and shape of the analytes. The retention time in the MCC and mobility in the IMS characterize the identity of the analyte. The intensity of the signal is a measure of the analyte's concentration.

Breath analysis was performed by IMS. The multi-capillary column/ion mobility spectrometry (MCC/IMS) device was custom designed by the Leibniz-Institute for Analytical Sciences (ISAS, Dortmund, Germany) (Figure 1). In brief, the MCC/IMS works as follows: A β-radiation source is used to provide reactant ions (protonated water clusters) by ionization of the available gas (synthetic air) in a ionization chamber. When a gas phase sample is introduced into the ionization chamber, proton transfer from the reactant ions to the analyte molecules takes place, thus forming protonated analyte monomer ions. The ions are accelerated in a weak electric field (330 V/cm). During their drift, the ions collide with the drift gas molecules moving in the opposite direction. The collision rate depends on the size and shape of the ions and leads to a resulting drift velocity characteristic for the analyte ion. A detailed description of the principles of ion mobility spectrometry was published previously [7]. The drift time of the ions is normalized to the drift length, the electric field, temperature, and pressure. The resulting inverse of the reduced ion mobility K_0_ is a measure for the identity of the analyte. The signal intensity is a measure for the analyte concentration. Signal intensity is measured in Volts, but normalized to the reactive ion peak and provided in arbitrary units (a.u.). A MCC is used for fast gas-chromatographic preseparation, thus allowing the analysis of complex, humid mixtures. The sample was gained from the main stream of the exhaled breath through a sample loop of 8 ml for 20 s to avoid memory effects. The obtained retention time (t_R_) of the MCC constituted an additional measure for the identification of the analyte.The experimental parameters of the instrument are presented in Table 3, further details are described in the literature [8].The software used for instrument control, data acquisition and evaluation of the 3-dimensional MCC/IMS data was developed at ISAS.

**Table 3:** Experimental parameters of the multi-capillary column ion mobility spectrometer (MCC/IMS) as used in the present study.

Parameter	Settings
Ionization source	β-radiation (^63^Ni, 550 MBq)
Drift length	12 cm
Drift field	330 V/cm
Grid opening time	300 µs
Drift gas	100 ml/min synthetic air
Carrier gas	150 mL/min synthetic air
Preseparation	MCC OV-5*, 20 cm, operated isothermal at 40°C

* Multichrom, Novosibirsk, Russia.

### Identification of analyte molecules by thermal desorption gas chromatography/mass spectrometry (TD-GC/MS)

The identification of analytes detected in MCC/IMS spectra is possible only by comparison with a data base of the characteristic mobility and retention time of analytes. Therefore, a further analytical method is required for the identification of unknown signals in the spectra. Random breath samples (2 l) were drawn on adsorption tubes (Tenax^R^, SKC Inc., USA) for later analysis using thermal desorption gas chromatography/mass spectrometry (TD-GC/MS)immediately after the MCC/IMS analysis of the same patient. TC-GC/MS was performed as published previously using an Agilent Technologies 6890N GC system connected with an Agilent Technologies 5973 mass-selective detector (MSD, Gerstel, Mühlheim, Germany) [9]. After evaluation of the GC/MS data and correlation with the unknown signals in the MCC/IMS spectra using an alignment procedure for the retention time of the GC and the MCC (alignment t_R_), a proposal for the analyte responsible for the MCC/IMS signal was made. The proposal was then validated by reference measurements carried out using a calibration gas generator (HovaCAL, IAS, Frankfurt, Germany). This procedure has been routinely performed at the ISAS for years. Thus, a database was established allowing a rapid identification of molecules underlying the MCC-IMS spectra. Only the unknown signals in the present study, required identification by TD-GC/MS.

### Analysis of spectral data

**Figure 2:**
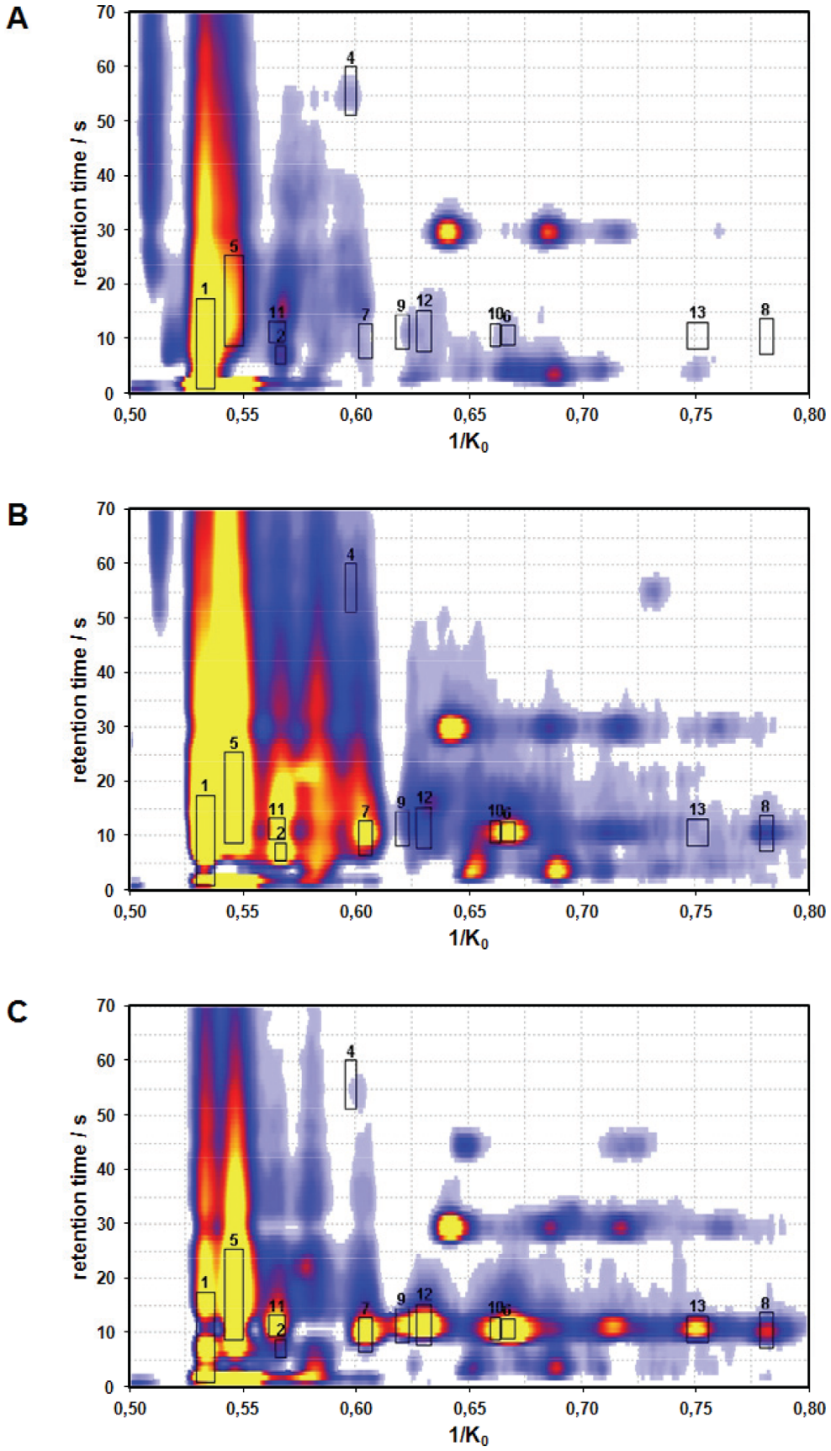
Representative multi-capillary column/ion mobility spectra (MCC/IMS) of breath samples. Breath sample of (A) a healthy adult, (B) an end-stage renal disease proband before and (C) after hemodialysis treatment. Areas of interest are marked and labeled by numbers. Substances corresponding to these numbers are given in Table 2. Signal intensity is coded by colours (yellow: very high; red high, blue: moderate, white: no signal).

Spectra of the MCC/IMS were presented as t_R_ over 1/K_0_ with a colour coding of signal intensity (Figure 2). Analytes with the intensity rank order “group A<B<C>D ” were selected by using software tools developed at ISAS (IPHEX, BB_IMSanalyse) for further evaluation [10], [11]. The algorithms that are used by these software tools to select peaks of interest are described in the literature [12]. Moreover, analytes that preferentially or exclusively occurred in patients undergoing hemodialysis were selected. Thirteen analytes were selected for further investigation by this procedure. TD-GC/MS was used in an attempt to identify these fourteen analytes. Those analytes, that could not be definitely identified, are presented as “1/K_0_−t_R_” in the following. The diagnostic performance of the substances in discriminating normal and impaired renal function was performed as described below.

### Statistics

Data are presented as mean ± standard deviation. Existence of significant intergroup differences in signal intensities was tested by univariate ANOVA. The individual groups were compared by two-sided two-sample Student's t-tests. P<0.05 was regarded significant. All statistical analyses were done using PASW Statistics 18.0 (SPSS Inc, Chicago, Illinois, USA).

## Results

**Figure 3:**
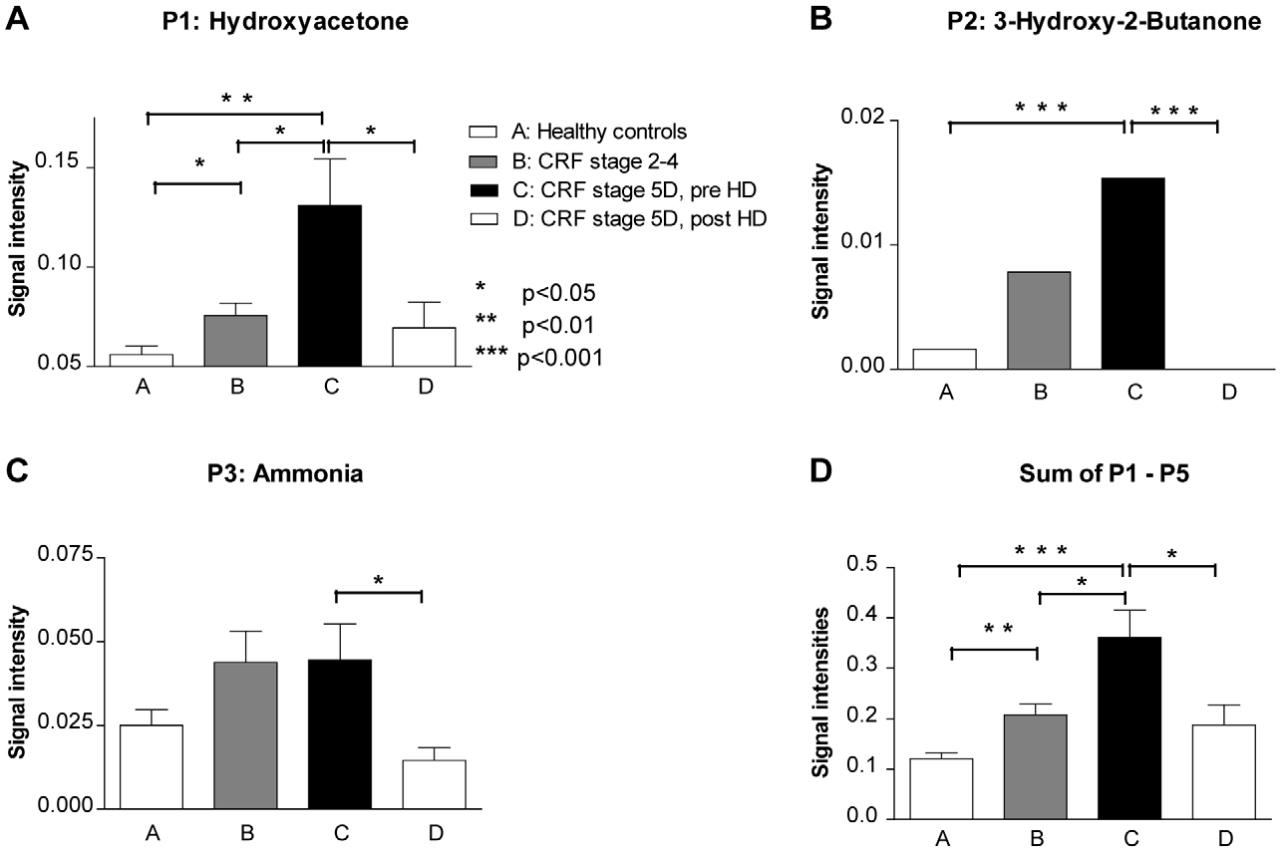
Signal intensities of exemplary analytes that accumulate with decreasing renal function and are eliminated by dialysis. Figures A–C present signal intensities of exemplary analytes P1–P3 and Figure D the sum of the signal intensities of the five analytes that accumulate with decreasing function and are eliminated by dialysis (P1–P5) in 28 healthy controls, 26 patients with chronic renal failure (CRF) stage 2–4 according to K/DOQI-criteria, 28 patients with end-stage renal disease (ESRD, CRF stage 5D) prior to and 22 after hemodialysis. Signal intensities were tested for statistical significance by two-tailed t-tests; *p<0.05, **p<0.01, ***p<0.001.

**Figure 4:**
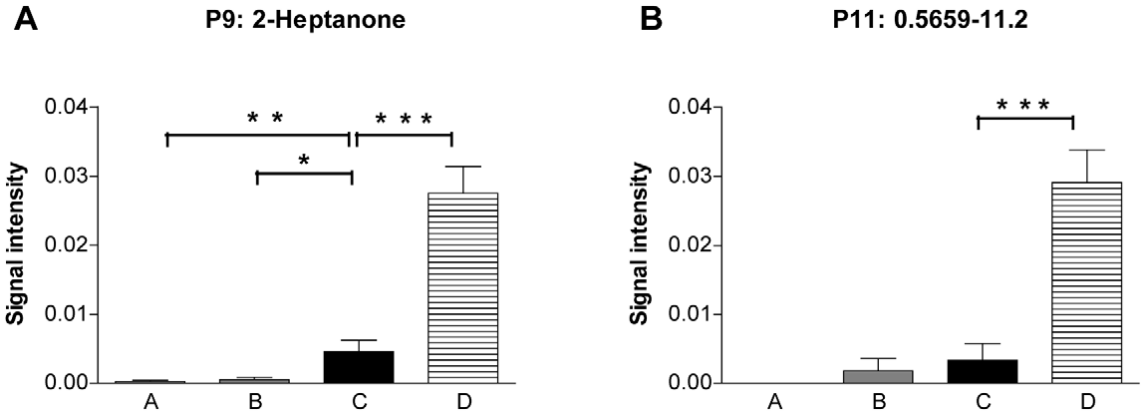
Signal intensities of exemplary analytes that accumulate during hemodialysis. Signal intensities of analytes P9 and P11 in 28 healthy controls, 26 patients with chronic renal failure (CRF) stage 2–4 according to K/DOQI-criteria, 28 patients with end-stage renal disease (ESRD, CRF stage 5D) prior to and 22 after hemodialysis. Signal intensities were tested for statistical significance by two-tailed t-tests; *p<0.05, **p<0.01, ***p<0.001.

Breath analysis was successfully performed by IMS in 28 adults with an eGFR ≥60 ml/min per 1.73 m^2^ (group A), 26 adults with chronic renal failure (CKD) corresponding to an eGFR of 10–59 ml/min per 1.73 m^2^ (group B), and 28 adults with end-stage renal disease (ESRD) undergoing renal replacement therapy (before and after hemodialysis corresponding to group C and D). A characterization of the study population is provided in Table 1. Spectra of healthy study participants and patients with ESRD differed markedly as visualized in Figure 2. Using the above mentioned software tools, thirteen analytes with apparent differences between healthy controls and ESRD patients were chosen for further analysis (Table 2). Seven of them were successfully identified by TD-GC/MS. These analytes fall into two categories:

1. The concentrations of five analytes increased with decreasing renal function and were reduced by hemodialysis (signal intensity group rank order “A<B<C>D”). Three of these peaks could be identified by TD-GC/MS (P1: hydroxyacetone, P2: 3-hydroxy-2-butanone, P3: ammonia) and two are unknown yet (P4: 0.5985–55.6, and P5: 0.5468–17.0). These analytes are displayed in Figure 2 (P1–5) and Figure 3.
2. There were eight analytes that preferentially or exlusively occurred in patients undergoing hemodialysis (group C and D only). Four of these analytes could be identified (P6: hepatanal, P7: 4-heptanone monomer, P8: 4-heptanonedimer, P9: 2-heptanone) and four remain elusive (P10: 0.6623–10.7, P11: 0.5659–11.2, P12: 0.6308–11.4, P13: 0.7517–10.6). These analytes are visualized in Figure 2 (P6–13) and Figure 4.

For the five analytes with the group rank order A<B<C>D, t-tests were used to compare the means of the signal intensities corresponding to the individual stages of renal disease and to compare the intensity before and after hemodialysis (Figure 3). All five analytes showed significant differences in signal intensity before and after dialysis. Hydroxyacetone revealed significant differences between all the individual stages of renal failure (p<0.05 each). In an attempt to optimize the diagnostic accuracy of the compounds in differentiating healthy subjects from those with impaired renal function, the sum of the five analytes with the rank order A<B<C>D was calculated. As presented in Figure 3D, the sum was 0.12±0.065 a.u. (group A), 0.21±0.11 a.u. (group B), 0.36±0.29 a.u. (group C), and 0.19±0.19 a.u. (group D). ANOVA analysis indicated a highly significant difference between the signal intensities of the different groups (p<0.001). T-test analysis showed that the signal intensity sum of “healthy” adults (eGFR≥60 ml/min per 1.73 m^2^) was significantly different from patients with an eGFR of 10–59 ml/min per 1.73 m^2^ (p 0.001), and from ESRD patients prior to dialysis (p<0.001) as presented in Figure 3D. Moreover, patients with an eGFR of 10–59 ml/min per 1.73 m^2^ were significantly different from ESRD patients prior to dialysis (p 0.01). The signal intensity was significantly reduced by hemodialysis (groups C and D, p 0.02).

**Figure 5:**
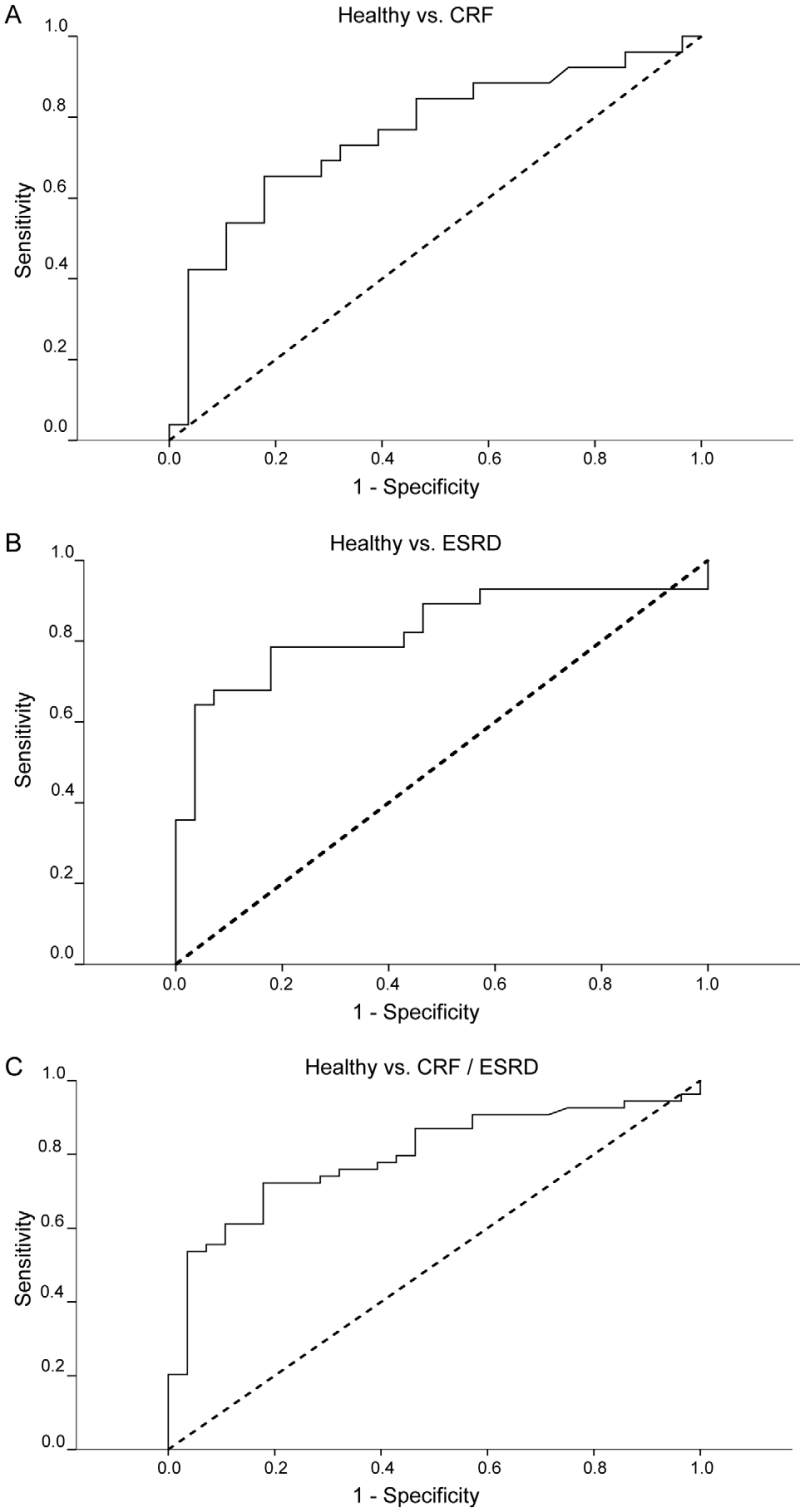
Receiver-operating-characteristic (ROC) curves to distinguish different stages of renal failure. ROC curves for the sum of the signal intensities of hydroxyacetone, hydroxy-2-butanone, ammonia, 0.5468–17.0, and 0.5985–55.6 in differentiating (A) healthy subjects and patients with chronic renal failure (CRF) corresponding to an eGFR of 10–59 ml/min per 1.73 m^2^ (AUC 0.76), (B) healthy subjects and patients with endstage renal disease (ESRD, AUC 0.83), and (C) healthy subjects and all patients with impaired renal function (CRF and ESRD; AUC 0.80).

ROC curves were built to assess the diagnostic performance of the sum of the signal intensities in distinguishing different stages of renal failure. As shown in Figure 5A, this value achieved an area under the curve (AUC) of 0.76 in differentiating healthy subjects from patients with impaired renal function corresponding to an eGFR of 10–59 ml/min per 1.73 m^2^ (Figure 5A) and an AUC of 0.83 to differentiate healthy and ESRD (Figure 5B). ROC curve for the differentiation of “healthy” (eGFR≥60 ml/min per 1.73 m^2^) and “ill” (eGFR<60 ml/min per 1.73 m^2^) provided an AUC of 0.80 (Figure 5C). The latter ROC curve revealed an optimal threshold of 0.15 a.u. to distinguish “healthy and ill”. This threshold provided a sensitivity of 72.2%, a specificity of 82.1%, a positive predictive value of 88.6%, and a negative predictive value of 60.5% in predicting an impairment of renal function.

Further signals with visually recognizable intergroup differences occurred in the breath of the study population. In the present proof of principle study, however, we limited the analysis to the above mentioned peaks for two reasons: First, we focused on those analytes with low intragroup variabilities. Second, some signals overlapped with others, thus precluding an accurate identification and quantification with the predefined settings of the IMS. A typical example of such analytes was dimethylamine and di-/trimethylamine, which were detected in the shoulder of the significantly more intense ammonia signal. Due to this overlap exact quantification was difficult and we refrained from including these compounds in the quantitative analysis.

## Discussion

The present work shows that breath analysis by ion mobility spectrometry is able to identify VOCs that are retained in uremia. Breath analysis revealed significant differences in the spectra of patients with and without renal failure. This “uremic fingerprint” encompasses several compounds that accumulate in renal failure and are eliminated by hemodialysis. Due to the lipophilic nature of the alveolocapillary membrane the retention molecules in the exhaled air are predominantly lipophilic. The current approach may be of clinical interest since it may reflect the retention level of lipophilic uremic retention molecules. As stated above, there is increasing evidence that lipophilic protein-bound toxins are responsible for several biochemical and functional alterations in uremia and are inadequately reflected by the kinetics of urea and creatinine [5].

In this proof of concept study, we found several volatile compounds that accumulated in renal failure and are eliminated by hemodialysis. Five of these analytes were prompted to an identification procedure by TD-GC/MS. We identified ammonia, 3-hydroxy-2-butanone and hydroxyacetone (Table 3). It has previously been demonstrated that the elimination of ammonia by hemodialysis can be monitored by measurement of ammonia in the breath [13], [14]. The authors showed that breath ammonia correlated with blood ureanitrogen and creatinine levels during hemodialysis and concluded that breath ammonia measurements could be used as a real-time surrogate measure of the retention status of patients with ESRD [13], [14]. In the present study an increasing impairment of renal function went along with an increase in breath ammonia concentration. In accordance with the above mentioned study, dialysis reduced the concentration of ammonia (Figure 3C). Ammonia is a compound of predominantly endogenous origin. 3-hydroxy-2-butanone (acetoin) is an organic compound generated primarily by bacteria and plants. Acetoin is used for the production of artificial flavors and is a natural ingredient of yoghurt, and several fruits and vegetables. Thus, 3-hydroxy-2-butanone in the breath may be of exogenous origin. In pathological situations, however, it may be generated endogenously as well: There are increased breath concentrations of 3-hydroxy-2-butanone in patients with tumors like lung or hepatocellular carcinoma [15]–[17]. According to Philipps et al., 3-hydroxy-2-butanone may be considered as an oxidative product of butane [17]. The production of 3-hydroxy-2-butanone therefore represents increased oxidative activity, which is regarded to be the reason for its predictive value in the detection of lung cancer [17]. It may be speculated that the pathological condition of renal failure leads to an endogenous production of 3-hydroxy-2-butanone as well. Independent of its origin the present data strongly indicate renal elimination. The compound is completely removed from the body by dialysis (Figure 3B).

Besides compounds that accumulated with increasing impairment of renal function there were nine substances that preferentially or exclusively occurred in patients undergoing hemodialysis, e.g. heptanal and the ketons 4-heptanone and 2-heptanone. Figure 4A illustrates the occurrence of 4-hepatone in the four groups. This distribution intuitively suggests that these substances are derivatives from the hemodialysis procedure itself. Indeed, heptanal is used in the production of paints. 4-Heptanone is a metabolite of diethylhexylphthalate, the most frequently used industrial emollient in synthetic materials. Phtalates are industrial chemicals used as plasticizers, softeners, adhesives or solvents and are used in hemodialysis tubing systems. 4-hepatone has been previously described in the plasma of hemodialysis patients as a degradation product of diethylhexylphthalate [18].

The present work shows that there are several volatile compounds that mirror the state of uremic retention. A pathophysiological role has been described for some of them. E.g., trimethylamine is associated with impaired brain function and is suggested to be associated with higher cancer rates in ESRD patients [4], [19]. The question arises whether the knowledge about this new class of uremic toxins may be of use in daily clinical medicine. As stated above, there is an ongoing search for markers of the retention niveau of protein-bound uremic toxins. Lipophilic volatile compounds may be a promising opportunity in this context. Second, breath analysis may be used as a screening method for renal failure. The non-invasive nature of this method may be of special interest for pediatric care. Third, this approach may be used for real-time monitoring of hemodialysis efficacy. The proof of principle has already been provided using ammonia [13], [14]. Comparing the elimination of ammonia (Figure 3C) with 3-hydroxy-2-butanone (Figure 3B), however, reveals that 3-hydroxy-2-butanone might be a more accurate marker: It is removed completely by hemodialysis. Hence, there are no re-filling effects from third compartments that impede the accuracy of current approaches to assess the efficacy of dialysis. Fourth, the technique has proven to be able to detect compounds derived from the hemodialysis extracorporal circuit. Therefore, it may be a helpful adjunct in the assessment of treatment linked toxicity.

The present study is limited by the fact that we identified only a subgroup of volatile compounds in uremic breath so far. The selection of the thirteen analytes that were forwarded to the attempt of TD-GC/MS identification was based on three aspects: 1) association to renal function or hemodialysis, 2) low intragroup variability, and 3) differentiability to other peaks. Comparison of the spectra of healthy subjects and patients with ESRD revealed many other analytes that accumulated in renal failure. The majority of these analytes were excluded from analysis for the third reason. In this proof of principle study we made use of a standard setting of the MCC/IMS and a limited number of subjects. An optimization of the settings – e.g. preseparation temperature, field strength, drift gas and carrier gas flow, preseparation and drift length – and a larger population of patients with renal failure will allow a more distinct separation of analytes that are now displayed very close to one another. Hariharan et al. recently demonstrated the potential of optimizing experimental parameters with regard to peak resolution [20]. Moreover, TD-GC/MS was not able to identify two analytes, potentially due to the low number of patients and low concentrations in the breath. The identification of the unknown analytes will be a major objective of larger studies in the future.

What are the next steps to transfer this promising method to clinical practice? Larger studies yielding at an optimization of MCC/IMS and GC/MS settings for volatile uremic compounds are required. An optimization of the settings will allow the detection and identification of more substances that are currently displayed too close to one another for separation. Moreover, the studies should include clinical signs, symptoms, and endpoints in order to define either a potential pathophysiological meaning or the reflection of clinical parameters of the detected substances. Finally, sequential breath analyses should be performed during hemodialysis and should be correlated to current standard procedures for the calculation of dialysis efficacy.

The present study shows that breath analysis by a combination of gas chromatography and ion mobility spectrometry can be used for the identification of volatile organic compounds retained in uremia. The combination of these techniques was of crucial relevance, since it provides a sensitive and rapid (10 min) measurement without a need of sample pretreatment. It may be speculated that the lack of an adequate technical solution like this is the reason why uremic breath has not been analyzed in a systematic manner so far. The present first global analysis of uremic breath shows that renal impairment induces a characteristic fingerprint of volatile compounds in the breath. Future studies have to elucidate whether the findings on this new class of uremic toxins can be transferred into clinical practice.
